# Identification of a Locus Conferring Dominant Susceptibility to *Pyrenophora tritici-repentis* in Barley

**DOI:** 10.3389/fpls.2020.00158

**Published:** 2020-02-28

**Authors:** Bohan Wei, Matthew J. Moscou, Kazuhiro Sato, Ryan Gourlie, Stephen Strelkov, Reem Aboukhaddour

**Affiliations:** ^1^ Cereal Pathology Lab, Agriculture and Agri-Food Canada, Lethbridge Research and Development Centre, Lethbridge, AB, Canada; ^2^ Department of Agricultural, Food and Nutritional Science, University of Alberta, Edmonton, AB, Canada; ^3^ The Sainsbury Laboratory, University of East Anglia, Norwich, United Kingdom; ^4^ Institute of Plant Science and Resources, Okayama University, Kurashiki, Japan

**Keywords:** tan spot, barley, race 5, quantitative trait locus mapping, Ptr ToxB, chlorosis

## Abstract

The fungus *Pyrenophora tritici-repentis* (*Ptr*) causes tan spot, a destructive foliar disease of wheat worldwide. The pathogen produces several necrotrophic effectors, which induce necrosis or chlorosis on susceptible wheat lines. Multiple races of *Ptr* have been identified, based on their ability to produce one or more of these effectors. *Ptr* has a wide host range of cereal and non-cereal grasses, but is known to cause damage only on wheat. Previously, we showed that *Ptr* can interact specifically with cultivated barley (*Hordeum vulgare* ssp. *vulgare*), and that the necrotrophic effector Ptr ToxB induces mild chlorosis in a highly selective manner when infiltrated into certain barley genotypes. In the present study, a barley doubled-haploid (DH) population was evaluated for reaction to *Ptr* race 5, a Ptr ToxB-producer. Then a comprehensive genetic map composed of 381 single nucleotide polymorphism (SNP) markers was used to map the locus conditioning this chlorosis. The F1 seedlings, and 92 DH lines derived from a cross between the resistant Japanese malting barley cultivar Haruna Nijo and the susceptible wild barley (*H.* vulgare ssp. *spontaneum*) OUH602 were inoculated with a conidial suspension of *Ptr* race 5 isolate at the two-leaf stage. The seedlings were monitored daily for symptoms and assessed for chlorosis development on the second leaf, 6 days after inoculation. All tested F1 seedlings exhibited chlorosis symptoms similar to the susceptible parent, and the DH lines segregated 1:1 for susceptible:resistant phenotypes, indicating the involvement of a single locus. Marker-trait linkage analysis based on interval mapping identified a single locus on the distal region of the short arm of chromosome 2H. We designate this locus *Susceptibility* to *P. tritici-repentis*1 (*Spr1*). The region encompassing this locus has 99 high confidence gene models, including membrane receptor-like kinases (RLKs), intracellular nucleotide-binding, leucine-rich repeat receptors (NLRs), and ankyrin-repeat proteins (ANKs). This shows the involvement of a dominant locus conferring susceptibility to *Ptr* in barley. Further work using high-resolution mapping and transgenic complementation will be required to identify the underlying gene.

## Introduction

*Pyrenophora tritici-repentis* (*Ptr*), an ascomycete fungus, is a necrotrophic pathogen causing tan spot, an important foliar disease of wheat. *Ptr* infects its primary wheat host (*Triticum aestivum* L. and *Triticum turgidum* L.) worldwide, and has been isolated from numerous graminaceous species including rye, barley, oat, bromegrass, and several prairie grasses that may function as secondary hosts for the pathogen (Ciuffetti et al., 2014; De Wolf et al., 1998). *Ptr* was first isolated and characterized from the grass species *Agropyron repens*, almost a century before it was identified as a pathogen of wheat (De Wolf et al., 1998). Grasses were, for a long time, considered as the primary host for this fungus, then both *A. repens* and *Triticum* sp. were regarded as its main hosts, explaining why the fungus was given its hyphenated name *P. tritici-repentis* (De Wolf et al., 1998). *Ptr* has a wide host range of cereal and non-cereal grasses on which the fungus can survive (Ali and Langham, 2015). The vast majority of research on tan spot has focussed on understanding the interaction of *Ptr* with its primary wheat host (reviewed in Ciuffetti et al., 2014). Early research explored, albeit in a descriptive manner, the interaction between *Ptr* and other hosts by defining the severity of symptoms, or the ability of the fungus to reproduce, and evaluated the pathogenicity of *Ptr* isolates collected from grasses on wheat (reviewed in De Wolf et al., 1998).

*Ptr* was found to colonize barley (*Hordeum vulgare* ssp. *vulgare*) saprophytically (Summerell and Burgess, 1988), or to cause moderate to severe damage on this species (Morrall and Howard, 1975; Postnikova and Khasanov, 1997). It also was reported that *Ptr* produced a host-specific toxin of low molecular weight and an acidic nature that could cause moderate chlorosis on barley (Brown and Hunger, 1993); however, that toxin was not characterized further or identified in any subsequent studies. More recently, *Ptr* was found to interact specifically with barley, with the interaction mediated by the chlorosis-inducing necrotrophic effector Ptr ToxB (Aboukhaddour and Strelkov, 2016). While the symptoms induced by *Ptr* on barley were weaker than those on wheat, and a higher concentration of Ptr ToxB was needed to induce chlorosis on the barley (Aboukhaddour and Strelkov, 2016; See et al., 2019), the specificity between *Ptr* and barley was evident, since chlorosis developed on certain barley genotypes but not on others (Aboukhaddour and Strelkov, 2016). Furthermore, infiltration of Ptr ToxB by itself induced chlorosis on the same barley genotypes rated as susceptible to the producing fungal isolate, but not on genotypes rated as resistant. Thus, susceptibility to the pathogen and sensitivity to the effector appear to be associated (Aboukhaddour and Strelkov, 2016).

Despite the milder chlorosis that was developed on some barley genotypes, the pathogen was able to invade susceptible and resistant barley to the same extent, with no considerable difference in the cytology of infection, nor in the amount of fungal biomass detected in tissues after infection (Aboukhaddour and Strelkov, 2016). *Ptr* can infect barley and wheat in similar way, with few exceptions. On barley, *Ptr* invaded the vascular bundle without causing any wilting or yellowing of the vascular tissues, and on resistant barley, the fungus advanced in the mesophyll layer without causing any symptoms (Aboukhaddour and Strelkov, 2016). This may indicate a high adaptability of *Ptr* on barley and suggests that specificity and pathogenicity in *Ptr* are not under the same genetic control (Aboukhaddour and Strelkov, 2016). Variation in the genetic control of pathogenicity and specificity have been reported for several fungal pathogens (Freeman and Rodriguez, 1993; Ware, 2006; Stukenbrock and Mcdonald, 2008).

*Ptr* can induce chlorosis on 13.5% of 74 tested Canadian barley cultivars, representing over 100 years of breeding barley in Canada (Aboukhaddour and Strelkov, 2016), and a high concentration of Ptr ToxB caused symptoms on all five barley genotypes tested from Australia (See et al., 2019). Nonetheless, the genetic basis of the interaction of *Ptr* with barley or with other non-wheat hosts has not been investigated. These hosts may not exhibit as severe damage as wheat in response to *Ptr*, but they provide additional sources for pathogen inoculum and survival, and may impact pathogen genetic variability and therefore disease management. *Ptr* follows an inverse gene-for-gene interaction with its wheat host, meaning that specific recognition between a pathogen effector and the host leads to disease development (Lamari et al., 2003). So far, three different necrotrophic effectors have been identified in *Ptr*, the necrosis inducing effector, Ptr ToxA, and the two chlorosis inducing effectors Ptr ToxB and Ptr ToxC. Each effector interacts with a specific dominant sensitivity gene in the wheat host, and host sensitivity to each effector is associated with susceptibility to the producing fungal isolates [reviewed in (Faris et al., 2013)]. Here, we hypothesized that the *Ptr*-barley interaction is specific and likely follows a one-to-one relationship. Although this interaction is subtle, and slight changes in incubation temperature after inoculation can cause shifts in the barley reaction from susceptible to resistant (Aboukhaddour and Strelkov, 2016).

Although Canadian or Australian barley exhibits sensitivity to Ptr ToxB, this effector is absent from the pathogen population in Australia, and rarely reported in North America. In these regions, Ptr ToxA is the predominant effector (Aboukhaddour et al., 2013). *Tsn1*, encoding a serine/threonine protein kinase, nucleotide binding, leucine-rich repeat protein, is the sensitivity gene to PtrToxA in wheat (Faris et al., 2010; Faris et al., 2013). Ptr ToxA-*Tsn1* interaction is the best characterized interaction for *Ptr*-wheat, and the remaining *Ptr* effector-wheat interactions await further characterization. Ptr ToxB-producing races of *Ptr* are common in the wheat centre of origin, and Ptr ToxB-producers were found mostly among isolates collected from durum wheat (Aboukhaddour et al., 2011). The aim of this study is to investigate the genetics of the *Ptr*-barley interaction to expand our understanding of the *Ptr* pathosystem in related species to wheat. quantitative trait locus (QTL) analysis for susceptibility to *Ptr* in barley was conducted using a doubled-haploid (DH) mapping population from a cross between a Japanese barley cultivar and wild barley.

## Materials and Methods

### Fungal Isolate and Inoculum Preparation

In this study, *Ptr* race 5 isolate Alg3-24 (Ptr-ToxB-producer) was used to inoculate barley genotypes. This is the same isolate that was used by Aboukhaddour and Strelkov (2016) to investigate the specificity of the *Ptr*-barley interaction. Alg3-24 was collected from durum wheat in eastern Algeria, and has been used as the standard *Ptr* race 5 isolate in several investigations on Ptr ToxB [reviewed in (Lamari and Strelkov, 2010)].

For inoculum preparation, a single-spore of Alg3-24 was recovered and grown on fresh V8-potato dextrose agar (V8-PDA) in a 9-cm diameter Petri plate (Lamari and Bernier, 1989b). Several mycelial plugs (0.5 cm in diameter) were then excised from the actively growing part (edge) of the colony, and transferred singly to 9-cm-diameter V8-PDA Petri plates. The fungal colonies were incubated in darkness for 5 days at room temperature, until the culture reached 4–5 cm in diameter, at which point sterile distilled water was added and the mycelium flattened with the bottom of flame-sterilized glass tube. The water was decanted and the plates were incubated under fluorescent light overnight at room temperature, following which they were transferred to the dark for 24 h at 15°C to induce sporulation. The sporulating cultures were then flooded with sterile distilled water and scraped gently with a sterilized wire loop to dislodge the conidia. The conidial suspensions were collected and the concentration of conidia was estimated with a Fuchs Rosenthal Counting Chamber (Hausser Scientific, Blue Bell, PA) and adjusted to 5,000 conidia ml^−1^. Two drops of Tween 20 (polyoxyethylene sorbitan monolaurate) were added per 100 ml of conidial suspension.

### Plant Material and Inoculation

A DH barley population consisting of 92 lines previously derived from a cross of Haruna Nijo (*H. vulgare* ssp. *vulgare*) x OUH602 (*H. vulgare* ssp. *spontaneum*) at Okayama University, Japan (Sato and Takeda, 2009), was evaluated for its reaction to the *Ptr* race 5 isolate Alg3-24. F1 generated plants also were inoculated. The first parent, Haruna Nijo is a two-row malting cultivar grown in Japan, and was rated resistant to the *Ptr* isolate Alg3-24. The second parent, OUH602 is a wild barley (*H. vulgare* ssp. *spontaneum*) genotype, and was rated as susceptible to this isolate. The hexaploid wheat genotype 6B662 (sensitive to Ptr ToxB and susceptible to *Ptr* race 5), and the two barley lines, Rivers and Norbert, both of which are six-row barley, were included as controls. Rivers and Norbert were rated as susceptible and resistant to *Ptr* isolate Alg3-24, respectively (Aboukhaddour and Strelkov, 2016). These two genotypes also were evaluated for their reaction to infiltration with the purified Ptr ToxB, and Rivers was rated sensitive, while Norbert was insensitive (Aboukhaddour and Strelkov, 2016).

All plant genotypes were planted in 10 cm-diameter plastic pots filled with Sunshine Potting Mix (W.R. Grace and Co., Fogelsville, PA) at a rate of eight seeds per pot. Each genotype was seeded in two independent pots, and the bioassay was replicated three times independently. The seedlings were maintained in growth cabinets at 20/18°C (day/night) with a 16 h photoperiod (180 mmol m^−2^ s^−1^) until inoculation at the 2–3 leaf stage. Briefly, the seedlings were inoculated with the conidial suspension (5,000 conidia ml^−1^), prepared as described above, until runoff using a sprayer connected to an airline (Lamari and Bernier, 1989a). Immediately following inoculation, the seedlings were transferred to a humidity chamber (>95% relative humidity) for 24 h. The plants were then transferred to growth cabinets with a 16 h photoperiod (180 mmol m^−2^ s^−1^) at 20/18°C (day/night) and 60% relative humidity. The seedlings were monitored daily for symptom development and were rated for symptom development at 6 days post-inoculation (dpi).

### Phenotypic Analysis

The 92 lines of the DH population, the parental genotypes Haruna Nijo and OUH602, and the Canadian control cultivars were screened in three experiments for symptom development at 6 dpi with *Ptr* race 5. Symptoms were rated on scale of 1 to 5 following Lamari and Bernier (1989b). In brief, infected plants were rated as follows: reactions 1 and 2 are resistant, and reaction 3 to 5 are susceptible. Reaction 1 (small dark spots without any surrounding chlorosis or necrosis); reaction 2 (small dark spots with a very small chlorotic halo at the site of infection); reaction 3 (small dark spots completely surrounded by a distinct chlorosis, with lesions not coalescing together); reaction 4 (small dark spots completely surrounded by a chlorotic zone with the lesions coalescing); and reaction 5 (small dark spots surrounded by a chlorotic zone, with almost all of the infected leaf chlorotic).

The phenotypic (disease severity) data were subjected to a χ^2^ test and ANOVA using the agricolae (v. 1.2–4) package of R (R v. 3.2.3) (R Core Team, 2018). For the ANOVA model, DH lines, parents, and controls were considered as fixed effects, while experiments were considered as random effects. The ANOVA was conducted across experiments. The least significance difference (LSD) and the coefficient of variation % (CV%) were calculated with agricolae.

### Quantitative Trait Locus Analysis

A genetic map was previously constructed using an oligo-nucleotide pooled assay (OPA) for high-throughput single nucleotide polymorphism (SNP) genotyping, and 381 SNP markers were selected that were distributed across all seven barley chromosomes (Sato and Takeda, 2009; Muñoz-Amatriaín et al., 2011). Interval mapping was performed using scanone (R/qtl) with the expectation–maximization (EM) method and a 2.0 cM step size. Experimental-wide threshold was determined using 1,000 permutations and controlled at α = 0.05.

### Comparison of *Tsc2* and *Spr1* Loci

*Tsc2* is the dominant locus conditioning sensitivity to Ptr ToxB in wheat, and is located on the short arm of 2B chromosome (Friesen and Faris, 2004). Chromosome 2B was retrieved from URGI (Appels et al., 2018) and a 5.8 Mb interval representing the *Tsc2* locus between markers XBE517745 and Xmag681 (Abeysekara et al., 2010) was extracted with Bedtools (Quinlan and Hall, 2010). Similarly, the barley genome was obtained from GeneBank (Mascher et al., 2017) and a 3.2 Mb interval representing the *Spr1* locus on barley 2H chromosome between genes HORVU2Hr1G004230 and HORVU2Hr1G006010 was extracted with Bedtools. The two extracted loci were aligned and visualized by large-scale genome alignment tool progressiveMauve (v. 20150226 build 10) with the default settings (Darling et al., 2010). The predicted genes within the *Spr1* locus were then compared in sequence identity to the *Tsc2* locus. Protein and coding sequences for wheat were retrieved from the JGI Genome Portal (IWGSC, 2014). BLASTP and BLASTN searches (e−10) were performed using predicted gene sequences from high confidence gene models in barley (Mascher et al., 2017) and wheat (IWGSC, 2014; Appels et al., 2018). Orthologs in the wheat chromosome 2B Tsc2 region were identified when percent identity was greater than 50% over a region covering 50% of the BLASTP query length.

## Results

### Phenotypic Analysis

**Figure 1:**
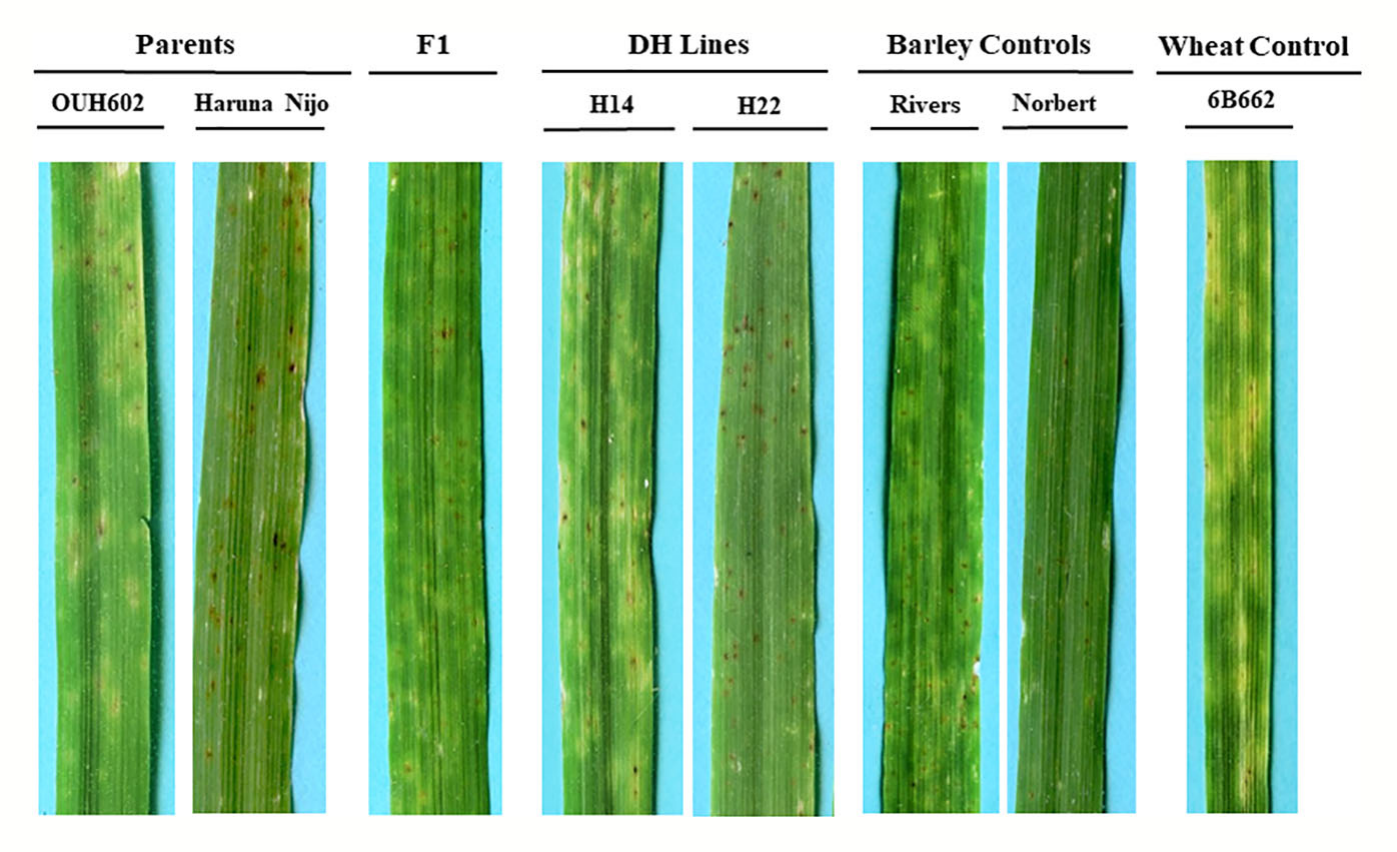
Reaction of barley to Ptr ToxB-producing race 5 isolate, Alg3-24. The two parents OUH602 and Haruna Nijo representing the susceptible and resistant reaction to race 5, respectively. The F1 plants exhibited the susceptible reaction. Two doubled haploid lines H24 and H22 representing a susceptible and resistant reaction, respectively. The barley controls, Rivers and Norbert were included as additional controls for susceptible and resistant reaction, respectively. The hexaploid wheat genotype 6B662 was also included as a susceptible wheat control.

**Figure 2:**
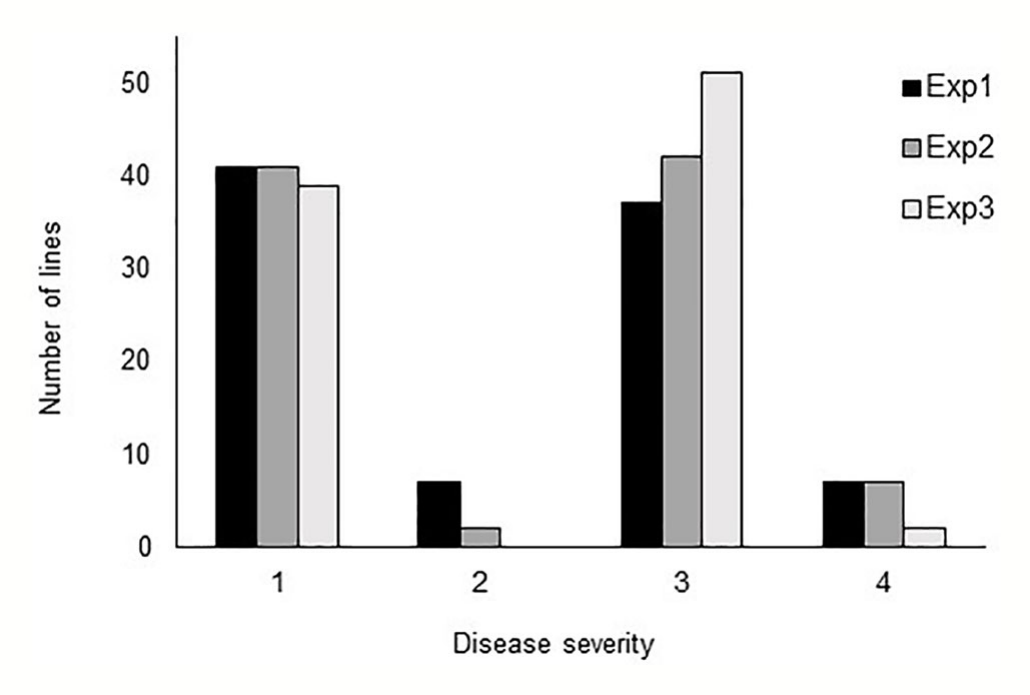
Barplot of the tan spot severity on barley DH lines. Disease severity was rated from 1 to 5, while 1 to 2 represent the resistance and 3 to 5 represent the susceptible reaction. The DH lines segregated in score 1 and score 3 into nearly 1:1 ratio.

In all three phenotyping experiments, Haruna Nijo scored as highly resistant, and OUH602 was rated as susceptible to *Ptr* isolate Alg3-24 (**Figure 1**). The disease severity of the DH lines ranged from 1 to 4, with a mean of 2.144 (**Table 1**). Among the parents and the controls, the disease severity of the resistant parent Haruna Nijo and the resistant control Norbert were scored as 1, while the mean disease severity of the susceptible parent OUH602 and the susceptible control barley cultivars ranged from 3 to 4 (**Table 1**). In the first run of the experiment, 48 (52%) of the DH lines were rated as resistant and 44 (48%) were rated as susceptible. In the second experiment, 43 (47%) lines were rated as resistant and 49 (53%) were rated as susceptible, while in the third experiment, 39 (42%) lines were rated resistant and 53 (58%) were rated susceptible (**Figure 2**). In all experiments, the segregating ratio susceptible:resistant was not significantly different from the expected 1:1 ratio at the 0.05 level of probability (χ² = 0.93) (**Table 2**). F1 plants of Haruna Nijo x OUH602 exhibited a chlorotic reaction similar to the susceptible parent OUH602, indicating that susceptibility to *Ptr* isolate Alg3-24 in this cross is dominant.

**Table 1:** Details of average and range of disease severity on 92 doubled-haploid lines, their parents, Haruna Nijo (*Hordeum vulgare* ssp. *vulgare*) and OUH602 (*H. vulgare* ssp. *spontaneum*), and control cultivars screened in three experiments after inoculation with *Pyrenophora tritici-repentis* race 5 isolate Alg3-24.

Experiments	Parent lines		Doubled-haploid lines			Controls			CV% (LSD)
	Haruna Nijo	OUH602	Min	Max	Mean	6B662	Rivers	Norbert	
1	1.0	3.0	1.0	4.0	2.096	4.0	3.0	1.0	10.48% (0.066)
2	1.0	3.0	1.0	4.0	2.163	4.0	3.0	1.0	
3	1.0	3.0	1.0	4.0	2.174	4.0	3.0	1.0	
Mean	1.0	3.0	1.0	4.0	2.144	4.0	3.0	1.0	

**Table 2:** Chi square table of doubled-haploid segregation in three experiments from a cross of Haruna Nijo and OUH602.

Experiments	Resistant lines	Susceptible lines	χ2 (d.f. = 1)
1	48	44	0.17
2	43	49	0.39
3	39	53	2.13
Total	130	146	0.93*

*Non-significant at 5% level of significance.

The LSD (p = 0.066), which was lower than the differences between parents, and the CV% (10.48%) (**Table 2**) showed that a large genetic effect contributed to disease resistance, and that the data were suitable for further analysis. The ANOVA (**Table 3**) indicated a highly significant genotype effect, as well as significant effect of experiments and experiments x genotype interactions, on tan spot disease severity.

**Table 3:** ANOVA of doubled-haploid barley lines from a cross of Haruna Nijo and OUH602 under the experiment effect, genotype effect and their interactions.

Source	d.f.	M. S.	*F*-value	Pr (> *F*)
Experiment (E)	1	0.1957	3.857	0.0525532**
Genotype (G)	91	3.2334	63.7449	< 2.2e−16***
(E x G)	91	0.1077	2.1240	0.0001892***
Error	92	0.0507		

**,***: significant at the 1 and 0.1% levels, respectively.

### Quantitative Trait Locus Analysis

**Figure 3:**
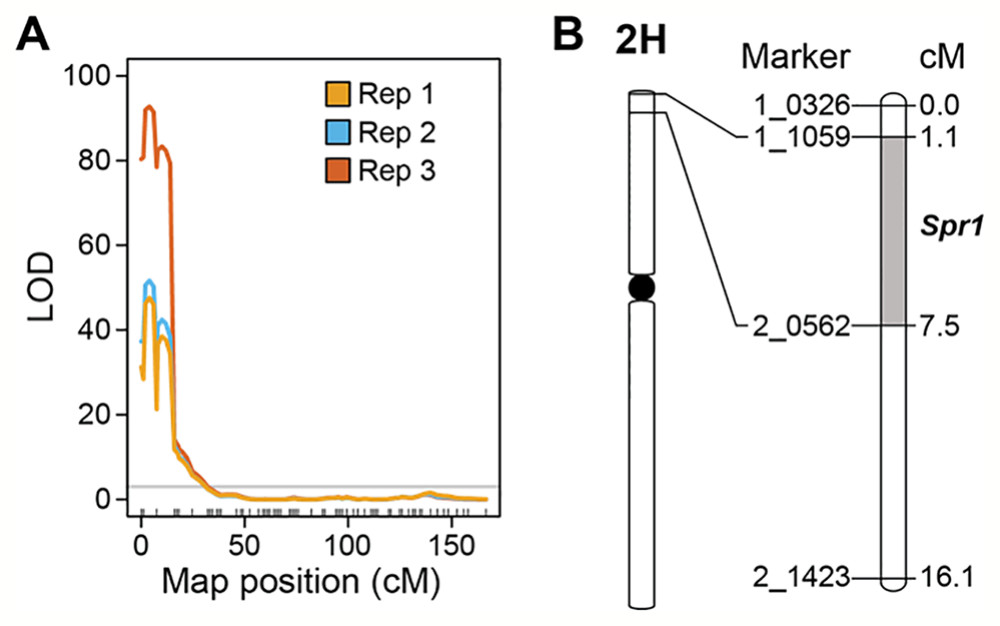
Mapping of *Spr1*. **(A)** Interval mapping based on replicated experiments. Permutation threshold of α = 0.05 is shown in grey. **(B)** Genetic interval encompassing the *Spr1* locus on chromosome 2H of barley.

Marker-trait linkage analysis based on interval mapping identified a single locus on the distal region of the short arm of chromosome 2H (**Figures 3A, B**). The QTL was flanked by SNP markers 1-1059 and 2-0562 in the genes HORVU2Hr1G004230 and HORVU2Hr1G006010, respectively (**Figure 3B**). LOD scores for this single QTL were 47.6 (experiment 1), 51.7 (experiment 2), and 92.8 (experiment 3) (**Figure 3A**). Susceptibility is the dominant trait, therefore we designate this locus *Susceptibility* to *P. tritici-repentis*1 (*Spr1*). On the barley physical map (Mascher et al., 2017), the interval ranges from 9.64 to 12.86 Mbp. The region encompassing the locus has 99 high confidence gene models, including membrane receptor-like kinases (RLKs), intracellular nucleotide-binding, leucine-rich repeat receptors (NLRs), and ankyrin-repeat proteins (**Supplementary Table 1**).

### Comparison of *Tsc2* and *Spr1*

The *Spr1* region contained 99 high-confidence candidate gene models (**Supplementary Table 1**). Multiple genome and protein alignments of the genes in *Spr1* and those in *Tsc2* showed that 73 of those genes are present as homologs in the wheat *Tsc2* locus based on BLASTP results (highlighted in the **Supplementary Tables 1** and **2**). An additional seven genes were identified as potential homologs based on BLASTN results (**Supplementary Tables 1** and **3**), these additional seven may be present only as non-coding sequences. Based on the 50-50 rule, 43 of the 73 homologous genes are present as orthologs in the *Tsc2* locus. Many of the predicted protein coding genes are involved in biotic and abiotic stress tolerance.

## Discussion

Genetic control of the *Ptr*-wheat interaction has been investigated for the last 50 years (reviewed in Faris et al., 2013). There is, however, no information on the genetics of the *Ptr* interaction with other hosts. *Ptr* is known to cause damage to wheat, but on other host species it is either non-pathogenic or causes moderate to severe symptoms (Morrall and Howard, 1975; Krupinsky, 1982; Postnikova and Khasanov, 1997; Ali and Francl, 2003). In one study, a number of *Ptr* isolates collected from 18 different grass species and cultivated barley were as aggressive on wheat cultivars as isolates recovered from wheat in the Northern Great Plains, and all the barley isolates tested were pathogenic on wheat (Krupinsky, 1992). Recently, an evidence of a specific interaction between *Ptr* and cultivated barley has been reported, and Ptr ToxB can act as a necrotrophic effector in barley as in wheat, albeit a higher concentration of this effector is needed to induce the chlorosis symptoms on barley (Aboukhaddour and Strelkov, 2016; See et al., 2019).

In this study, susceptibility to *Ptr* in barley was mapped to a single locus. The DH lines segregated in a 1:1 susceptible:resistant ratio following inoculation with *Ptr* race 5, and mapped to the short arm of chromosome 2H in barley. Moreover, F1 plants exhibited a chlorotic reaction similar to the susceptible parent OUH602, indicating that susceptibility to *Ptr* in this cross is dominant. Although the susceptible parent in this study was a wild barley, however, susceptibility in cultivated barley is also dominant. The F1 plants generated from a reciprocal crosses between two cultivated barley genotypes Rivers and Norbert, a susceptible and resistant lines, respectively, were susceptible to *Ptr* race 5 tested here (data not shown). This confirms that an inverse gene-for-gene model (Ellingboe, 1976), which mimics the *Ptr*-wheat interaction, is involved in the *Ptr*-barley interaction. This is the first genetic study on the interaction of *Ptr* with a secondary host, which will contribute to a greater understanding of the evolution of the *Ptr* pathosystem.

*Ptr* is not recognized as a barley pathogen and, indeed, in this study like in previous ones, we noted that the chlorosis on barley and wild barley was moderate and less intense than on susceptible wheat. Moreover, there was variation in the severity of the chlorosis that developed on the DH lines tested in this study (1 to 4 on a scale of 1 to 5), and on various barley genotypes in previous studies (Aboukhaddour and Strelkov, 2016; See et al., 2019). That may explain the wide range of symptoms described on barley in earlier studies by various groups (Morrall and Howard, 1975; Krupinsky, 1982; Summerell and Burgess, 1988; Brown and Hunger, 1993; Postnikova and Khasanov, 1997). This also suggests the presence of additional effectors produced by *Ptr*, which may contribute to the variation in symptom development on barley genotypes. Moreover, it was noted that the temperature after inoculation had a significant effect on symptom development, with declines or increases in the incubation temperature resulting in shifts in the host interaction from susceptible to resistant (Lamari and Bernier, 1994; Aboukhaddour and Strelkov, 2016). Therefore, establishing a consistent temperature for phenotype evaluation is critical.

The chromosomal region where the single locus was identified in this study encompasses 99 high confidence gene models, including genes from gene families known to be involved in plant immunity such as membrane receptor-like kinases (RLKs), intracellular nucleotide-binding, leucine-rich repeat receptors (NLRs), and ankyrin-repeat proteins (ANKs) (**Supplementary Table 1**). Multiple genome and protein alignments of the genes in *Spr1* and those in *Tsc2* showed the presence of 43 orthologous genes, and many of these genes have predicted function in abiotic and biotic stress tolerance. However, the exact identity and function of the gene mediating *Ptr*-barley interaction is unknown and cannot be predicted based on this information.

It is hypothesized that necrotrophic pathogens can utilize host resistance mechanisms for biotrophic fungi to their benefit, for example by proliferating in dead tissue resulting from the hypersensitive reaction and triggered by a host resistance gene (Shi et al., 2016). In wheat, *Tsn1* confers sensitivity to *Ptr* and susceptibility to Ptr ToxA-producing isolates. *Tsn1* is structurally related to plant disease resistance genes and includes serine/threonine protein kinase (S/TPK) and nucleotide-binding-leucine-rich repeat (NLR) domains (Faris et al., 2010). Interestingly, barley *Rpg5* stem rust resistance gene encodes a NB-LRR-S/TPK (Brueggeman et al., 2008), although these two genes encode two unrelated proteins (Faris et al., 2010). The interaction between a necrotrophic effector and a dominant sensitivity gene that is structurally similar to a typical biotrophic pathogen resistance gene is not unique and has been reported in other pathosystems (Shi et al., 2016).

*Ptr* is considered as new pathogen of wheat (Friesen et al., 2006), and it was suggested to have evolved on wild grasses prior to a host jump onto wheat (Strelkov and Lamari, 2003). On grasses, the race structure of *Ptr* is different from that on wheat. For example, while the non-pathogenic *Ptr* race 4 appears to be predominant on grasses, it is almost absent on wheat (Ali and Francl, 2003). Nevertheless, *Ptr* race 4 does carry the *toxb* gene, which is a homolog of *ToxB*, the Ptr ToxB-coding gene (Strelkov et al., 2006). The sequences of *ToxB* and its homolog in *Ptr* race 4 exhibit 86% similarity over the length of the open-reading frame (ORF) (Martinez et al., 2004; Strelkov et al., 2006). *ToxB*-like sequences are also found in race 3 isolates of *Ptr*, other species of the genus *Pyrenophora*, and even other genera of the *Pleosporacea* (Martinez et al., 2004; Strelkov et al., 2006; Andrie et al., 2007). Isolates of *Pyrenophora bromi*, a sister species to *Ptr* causing brown spot of brome grass, has several *ToxB*-like sequences (termed Pb ToxB) with coding regions having 89% similarity to *ToxB* (Andrie et al., 2008; Andrie and Ciuffetti, 2011). However, none of the heterologously expressed Pb ToxB proteins induced symptoms on brome grass, while they did cause chlorosis on *ToxB*-sensitive wheat genotypes (Andrie and Ciuffetti, 2011).

Several leaf spot causing pathogens of cereals or grasses share the same necrotrophic effectors or homologous coding gene sequences. Similar to *ToxB*, a homolog of the *ToxA* gene, which encodes Ptr ToxA, is found in *Bipolaris sorokiniana*, a pathogen infecting both wheat and barley (Mcdonald et al., 2018). Another *ToxA* homolog is also present in the maize pathogen *Cochliobolus heterostrophus* (Lu et al., 2015), and an identical *ToxA* sequence is present in the wheat pathogen, *Parastagonospora nodorum* (Friesen et al., 2006). Parallel to the presence of one effector or its homologs in various necrotrophic pathogens, related or unrelated plant genes conditioning sensitivity to one effector can exist in various host species, and these genes may condition multiple interactions with various plant pathogens (Lorang et al., 2007). The *LOV1* gene in *Arabidopsis* confers sensitivity to victorin, which is a secondary metabolite effector produced by the pathogen *Bipolaris victoriae* that devastated oat in the 1940s. *LOV1* belongs to the NLR class of resistance genes (Lorang et al., 2007). Similarly, the *Pc* gene in sorghum, which confers susceptibility to *Periconia circinata* and its Pc-effector, encodes an NLR (Nagy and Bennetzen, 2008).

The presence of *ToxB*-like sequences and non-functional homologs of Ptr ToxB in several species within two fungal orders (Dothideomycetes and Sordariomycetes) (Ciuffetti et al., 2014) remains unexplained. Why do these species code for what appear to be non-functional proteins? Ptr ToxB, like the other *Ptr*-necrotrophic effectors, does not appear to control any essential biological function in the fungus (Strelkov and Lamari, 2003). On wheat, Ptr ToxB interacts with a dominant sensitivity gene *Tsc2* on the short arm of the wheat chromosome 2B (Friesen and Faris, 2004). The exact mode of action to Ptr ToxB is not yet known, but treatment with this effector does cause chlorophyll photooxidation and an inhibition of photosynthesis (Lamari and Strelkov, 2010); this ultimately results in the development of chlorosis in wheat, similar to the symptoms observed here on cultivated and wild barley. On wheat, Ptr ToxB plays a considerable role in disease development, contributes to quantitative variation in the virulence of *Ptr*, and may influence development of fungal appressoria (Amaike et al., 2008; Aboukhaddour et al., 2012). Perhaps there are additional roles for Ptr ToxB and its various homologs that explain their presence in a wide range of fungal species, and which may also help to explain the interaction of *Ptr* with its secondary hosts.

## Data Availability Statement

All datasets generated and analyzed for this study are included and cited in the article/**Supplementary Material**.

## Author Contributions

BW performed most of the work in this manuscript and drafted the first version. KS developed the DH population and F1 plants, MM developed SNP markers and performed QTL mapping, RG performed sequence analysis of *Tsc2* and *Spr1* loci. SS, MM, and RA conceived the experiment and RA closely supervised the work. All authors reviewed, edited and contributed to this manuscript.

## Funding

Funding from University of Alberta, Alberta Wheat Commission and Saskatchewan Wheat Development Commission to BW, Agriculture and Agri-Food Canada and Alberta Wheat Commission and Saskatchewan Wheat Development Commission to RA, and Biotechnology and Biological Sciences Research Council (BB/P012574/1) and Gatsby Foundation to MM. The funding bodies were not involved in the design of the experiments and collection, analysis and interpretation of data, nor in the writing of this manuscript.

## Conflict of Interest

The authors declare that the research was conducted in the absence of any commercial or financial relationships that could be construed as a potential conflict of interest.
